# Dr. Frederick A. Levy

**Published:** 1893-05

**Authors:** Chas. A. Meeker

**Affiliations:** Newark, N. J.


### DR. FREDERICK A. LEVY.

Dr. Frederick A. Levy, aged forty-two years, was buried March 23, 1893, at Rosedale Cemetery, Orange, New Jersey.

Frederick Arthur Levy was born in Richmond, Virginia, and received a collegiate education in the College of Mobile, Alabama, after which, coming North, he entered the dental office of E. Parmly Brown, remaining there a year. He then entered the Baltimore College of Dental Surgery, and graduated in 1873. He settled in Orange, New Jersey, in 1874, and during the Centennial year became a member of the New Jersey State Dental Society at the meeting held at Atlantic City in 1879. He was subsequently elected President of the Society. Serving one year in this office, he was elected on the Board of Examiners. Upon the passage of the new law of the State Board of Registration and Examination in Dentistry, he was made the President of the Board, and held this office at his death. Dr. Levy was ex-President of the Central Dental Association of Northern New Jersey, Vice-President of the American Academy of Dental Surgery of New Jersey, Secretary and Treasurer of the National Board of Dental Examiners, Corresponding Secretary of the American Dental Association, and held the positions of chairman of the Registration and Finance Committees of the State for the World's Columbian Dental Congress.

To one who was more closely associated with him than any other member in furthering the cause of dental education and placing the profession on a high standard in New Jersey, no tribute that the writer can pay is adequate to the case. He never labored selfishly for power or professional advertising, but with a high sense of honor everything was done for the credit of the Association. He abhorred shams, and against these was outspoken and bitter in his denunciation. Everything done in the societies was by him performed for the glory of the organizations. His time, money, and voice were always at their service, and his unselfish work was recognized and acknowledged by all the members. Any office in their gift was at his disposal, and his advice was listened to with great respect.

A young daughter thirteen years of age survives him.

He possessed a modest library on dental matters, which no doubt will eventually be in the possession of the society, to be known as the "Levy Library."

The society and the profession have lost a valued member, and his intimates a true friend.

Chas. A. Meeker, D.D.S.

Newark, N. J.

RESOLUTIONS ON THE DEATH OF FREDERICK A. LEVY, D.D.S.

Whereas, In the death of Frederick Arthur Levy, D.D.S., our associate and valued member of thirteen years standing of the Central Dental Association of Northern New Jersey, the society has lost one of its most worthy and honored members, a man of sterling professional feeling, generous impulses towards his friends, and desire for the advancement of this society, therefore be it

*Resolved*, That the above expression of our regard and regret be placed upon the minutes of our society, and a copy forwarded to his surviving relatives and to the leading dental journals for publication.

Charles A. Meeker, D.D.S.,
George E. Adams, D.D.S.,
William L. Fish, D.D.S.,
*Committee.*

